# Grading Challenges and Prognostic Insights in Chromophobe Renal Cell Carcinoma: A Retrospective Study of 72 Patients

**DOI:** 10.3390/medicina60060996

**Published:** 2024-06-18

**Authors:** Dimitrios Papanikolaou, Ioannis Sokolakis, Kyriakos Moysidis, Nikolaos Pyrgidis, Mattheos Bobos, Soultana Meditskou, Konstantinos Hatzimouratidis

**Affiliations:** 1 Second Department of Urology, Medical School, Aristotle University of Thessaloniki, 541 24 Thessaloniki, Greece; 2 Department of Urology, University Hospital, LMU Munich, 80539 Munich, Germany; 3 Department of Biomedical Sciences, School of Health Sciences, International Hellenic University, 570 01 Thessaloniki, Greece; 4 Laboratory of Histology and Embryology, Medical School, Aristotle University of Thessaloniki, 541 24 Thessaloniki, Greece

**Keywords:** renal cell carcinoma, chromophobe, prognosis, nephrectomy

## Abstract

Chromophobe RCC (ChRCC) carries the best prognosis among all RCC subtypes, yet it lacks a proper grading system. Various systems have been suggested in the past, causing much controversy, and Avulova et al. recently proposed a promising four-tier grading system that takes into consideration tumor necrosis. Dysregulation of the mammalian target of the rapamycin (mTOR) pathway plays a key role in ChRCC pathogenesis, highlighting its molecular complexity. The present retrospective study aimed to evaluate the prognostic factors associated with a more aggressive ChRCC phenotype. *Materials and Methods:* Seventy-two patients diagnosed with ChRCC between 2004 and 2017 were included in our study. Pathology reports and tissue blocks were reviewed, and immunohistochemistry (IHC) was performed in order to assess the expressions of CYLD (tumor-suppressor gene) and mTOR, among other markers. Univariate analysis was performed, and OS was assessed using the Kaplan–Meier method. *Results:* In our study, 74% of patients were male, with a mean age of 60 years, and the mean tumor size was 63 mm (±44). The majority (54%) were followed for more than 10 years at intervals ranging between 44 and 222 months. The risk of death was significantly higher for patients that were classified as Grade 4 in the Avulova system (HR: 5.83; 95% CI, 1.37–24.7; *p*: = 0.017). As far as the IHC is concerned, mTOR expression was associated with an HR of 8.57 (95% CI, 1.91–38.5; *p* = 0.005), and CYLD expression was associated with an HR of 17.3 (95% CI, 1.57–192; *p* = 0.02). *Conclusions:* In our study, the Avulova grading system seems to be positively correlated with OS in patients diagnosed with ChRCC. Furthermore, an elevated mTOR expression also shows a negative correlation with OS, whereas an elevated CYLD expression does not seem to exert a protective role. However, because only a small proportion (4.2%) of our patients died due to ChRCC, despite the long follow-up period, the results must be interpreted with caution. Further research is needed to validate our findings.

## 1. Introduction

Chromophobe renal cell carcinoma (ChRCC) accounts for 5–7% of all RCC cases, thus making it the third most common subtype. It is divided into the following two separate morphologic variants: classic and eosinophilic [1]. In the latest edition of the World Health Organization (WHO) Classification of Tumors of the Urinary and Male Genital Systems, a molecular-driven classification of renal tumors was presented in addition to morphology-based renal tumors, creating a new category of oncocytic tumors to characterize those in the gray zone between oncocytoma and ChRCC [2]. Both ChRCC and oncocytoma originate from the intercalated cells of the collecting ducts in contrast to the other RCC subtypes, underlining the fact that ChRCC is a separate disease entity, sharing little cell lineage or genomic characteristics with them [3,4]. As a result, the clinical significance of common grading systems that are applied to other RCC subtypes, such as the WHO/ISUP (International Society of Urological Pathology) nucleolar system or the older Fuhrman grading system, could not be shown for ChRCC [5,6].

The need for an acceptable grading system has always existed, even though ChRCC carries the best prognosis among RCC subtypes. The 5- and 10-year disease-free survival reach 83.9% and 77.9%, respectively [7]. Given that a small but considerable portion is at risk of progression, numerous grading systems have been proposed. In this regard, Paner et al. suggested a three-tier system in 2010, while Ohashi et al. proposed a simplified two-tier grading system [8,9]. As tumor necrosis has been proven to be an independent predictor of cancer-specific survival in various studies, Avulova et al. recently proposed a four-tier grading system that includes tumor necrosis to improve prognoses, with very promising results [8,10,11,12,13].

As previously stated, ChRCC is histologically and molecularly different from the rest of the RCC subtypes [3,4]. In investigating its molecular mechanisms, it was found that the mTOR (Mammalian Target of Rapamycin) signaling pathway plays an important role in the disease, being frequently dysregulated not only in sporadic ChRCC cases but also in Birt–Hogg–Dube (BHD) syndrome, where patients tend to develop multiple bilateral ChRCC, among other clinical features [14,15]. Moreover, mutations in the mTOR signaling pathway are associated with poorer clinical outcomes [16]. As a result, the efficacy of mTOR inhibitors has been investigated in metastatic ChRCC cases, occasionally showing a good response to therapy [17].

Another gene that has been focused on regarding its roles in various types of neoplasias is the cylindromatosis gene cyld, which encodes the CYLD protein that functions as a deubiquitinase [18]. Its main regulatory target is nuclear factor kappaB (NF-kappaB), a transcription factor that promotes cell survival and oncogenesis, also interfering with other signaling pathways, such as tumor growth beta (TGF B) and c-Jun NH2-terminal kinase (JNK) [19,20]. It exerts its role by preventing the activation of the NF-kappaB pathway, thus acting as a tumor suppressor [19]. There is evidence of a tumor-suppressing role for CYLD in ccRCC, supported by the finding that Cyld mRNA was significantly downregulated in ccRCC samples in a pilot study; this is reflected in lower immunoreactive scores in immunohistochemistry (IHC) [21].

Here, we worked retrospectively with a large series of 72 ChRCC patients that were enriched with metastatic cases in order to validate the systems previously mentioned and determine whether some pathology features are associated with more aggressive behavior.

## 2. Materials and Methods

Approval of the study was granted by the Committee for Bioethics and Ethics of Aristotle, University of Thessaloniki (protocol no. 7389.20/04/2021). Informed consent was obtained from all subjects involved in the study. According to the eligibility criteria, patients could be included if they had undergone partial or radical nephrectomy with curative intent and were diagnosed with ChRCC, irrespective of their age. The exclusion criterion was the presence of different histological subtypes. We collected tumors from two different hospitals that serve as tertiary referral centers (Papageorgiou General Hospital of Thesssaloniki and Georgios Gennimatas General Hospital of Thessaloniki). In total, 72 patients fulfilled our selection criteria.

Pathology reports were examined in retrospect. More specifically, a surgical pathologist (AP) with expertise in renal pathology assessed all hematoxylin and eosin (H&E)-stained sections from the tissue blocks to confirm a diagnosis of ChRCC. Formalin-fixed paraffin-embedded (FFPE) tissue blocks were then chosen for further analyses by immunohistochemistry (IHC).

Clinical features (presentation, age, sex, laterality, year of operation, and type of nephrectomy) and gross pathology characteristics (greatest dimension and focality) were evaluated from the pathology reports and documented accordingly. Following this, each patient was evaluated by two separate pathologists (AP and MB) for the following pathology features: histology subtype, TNM T stage, presence of necrosis or sarcomatoid change, lymphovascular invasion, or positive surgical margins. Consequently, each case was graded according to the Paner and Avulova grading systems [8,13].

**Figure 1:**
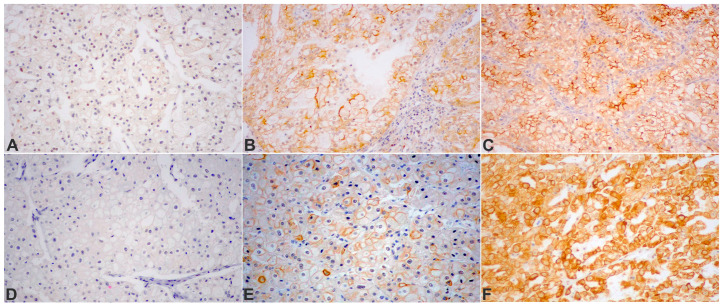
Protein expression detected by IHC in tissue microarrays of chromophobe RCC cases: (**A**) CYLD (CYLD lysine 63 deubiquitinase), absent in neoplastic cells; (**B**) CYLD, moderately diffuse and, in a fraction of the cells, strong membranous positivity; (**C**) CYLD, with focally strong membranous immunoreactivity; (**D**) ChRCC, completely negative for phospho-mTOR^Ser2448^ protein expression; (**E**) phospho-mTOR^Ser2448^, mild, moderate, and strongly diffuse, mainly membranous staining in tumor cells; (**F**) strong diffuse membranous and cytoplasmic expression of phospho-mTOR^Ser2448^ in the tumor cell population. DAB was used as a chromogen. Original magnification: ×200.

With respect to the IHC staining, it was conducted on 2.5 mm thick sections that were cut from the FFPE blocks. After completing deparaffinization and rehydration, the analytical phase of the IHC was carried out using an automated immunostaining platform (Bond Max II, Leica, Germany). In order to evaluate their expressions in the ChRCC tumors, the following markers were utilized in the IHC: CYLD, mTOR, and ki67. The rabbit polyclonal anti-CYLD antibody (dilution: 1/300, Sigma-Aldrich, St. Louis, MO, USA, SAB4200060) was applied for the detection of CYLD protein, and DAB was utilized as a chromogen. As previously described, a semiquantitative evaluation of the CYLD expression was calculated as a product of the staining intensity and quantity of immunoreactive tumor cells scores [22]. In particular, the staining quantity was classified as follows: 0 = no expression; 1 = positivity in less than 1% of cells; 2 = positivity in 1–9%; 3 = positivity in 10–50%; and 4 = positivity in more than 50% of cells. The staining intensity was defined as 0 = negative, 1 = low, 2 = medium, and 3 = high. As a final step, the intensity of the positive staining and the quantity (number of stained cells) were multiplied in order to obtain the final immunoreactive score (IRS), which ranged from 0 to 12. If the final IRS was ≥6, this was considered a high CYLD expression. Regarding the Ki67 marker, which was used to assess tumor proliferation, its expression was defined as low (<20%) or high (≥20%) based on the percentage of stained/unstained nuclei from the tumor area. Finally, for the phospho-mTOR protein expression, a semiquantitative assessment was conducted, as previously described for the CYLD marker. If the final IRS was ≥4, this was considered as high expression. Images of the CYLD and mTOR IHC staining are presented in Figure 1.

The continuous variables are summarized as means with the standard deviation (SD) and were compared with the *t*-test. Accordingly, the categorical variables are summarized as proportions and were compared with the chi-squared test. All parameters were visually assessed for normality using histograms and statistically with the Shapiro–Wilk test. Kaplan–Meier curves with the log-rank test were used to evaluate the overall survival. The effects of clinical and pathological parameters on the overall survival were also evaluated through a univariate Cox regression analysis. We estimated the hazard ratios (HRs) with 95% confidence intervals (CIs) for every survival outcome, and a two-sided *p*-value less than 0.05 was accepted as statistically significant. R statistical software (version 3.6.3) was the statistical program used for the analyses.

## 3. Results

The characteristics of the 70 patients that were included in our study are shown in Table 1. All procedures were carried out between 2004 and 2017, thus allowing for follow-up intervals that ranged between 44 and 222 months from surgery, with the majority (84%) followed for more than 5 years, and more than half (54%) followed for more than 10 years. The majority of the included patients were males (74%) with a mean patient age of 60.3 years. Tumors ranged in size from 10 to 230 mm (mean: 62.6 ± 44.0) with approximately two-thirds having undergone radical nephrectomy (69%).

Among our patients, 40 (57%) had ChRCC Grade 1, 23 (33%) Grade 2, and 7 (10%) Grade 3 tumors, as proposed by Paner et al. [8]. Using the newer scheme proposed by Avulova et al., 40 (57%) had Grade 1, 15 (21%) Grade 2, 8 (11%) Grade 3, and 7 (10%) Grade 4.

A total of 15 patients from our cohort died during their follow-up, including 3 who died from ChRCC. One of those three patients was already metastatic at the time of the operation and died two months later (graded as Paner Grade 2 or Avulova Grade 3). The other two patients were classified as Grade 3 by Paner or Grade 4 by Avulova. The rest of the patients died due to factors not related to ChRCC.

Univariate analysis of clinical parameters associated with death showed that only age (*p* = 0.014) and the type of operation (radical/partial nephrectomy) (*p* = 0.018) were significantly associated with worse outcomes. Regarding the assessed histopathology parameters, mTOR staining (*p* = 0.007) and CYLD IRS (*p* = 0.004) were significantly associated with worse OS. In our study, the presence of necrosis, sarcomatoid change, T stage, or positive surgical margins were not associated with a worse clinical outcome. The results of the univariate analysis are shown in Table 2 and Table 3.

**Figure 2:**
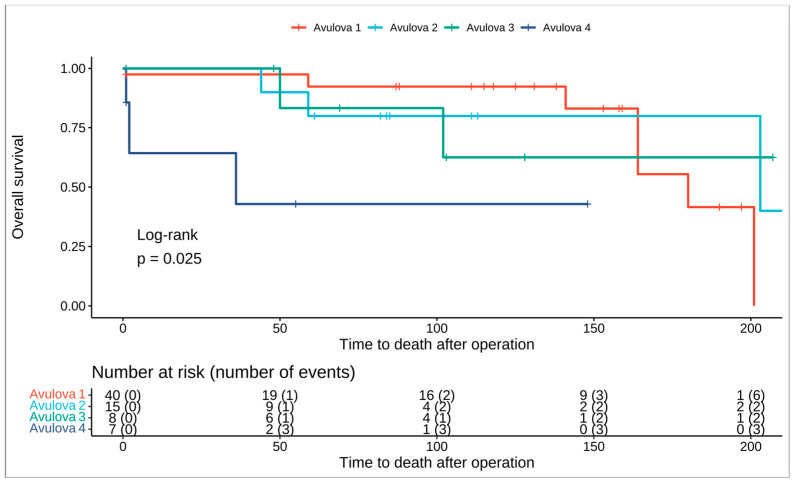
Kaplan–Meier curve showing overall survival for the Avulova 4-tier grading system.

**Figure 3:**
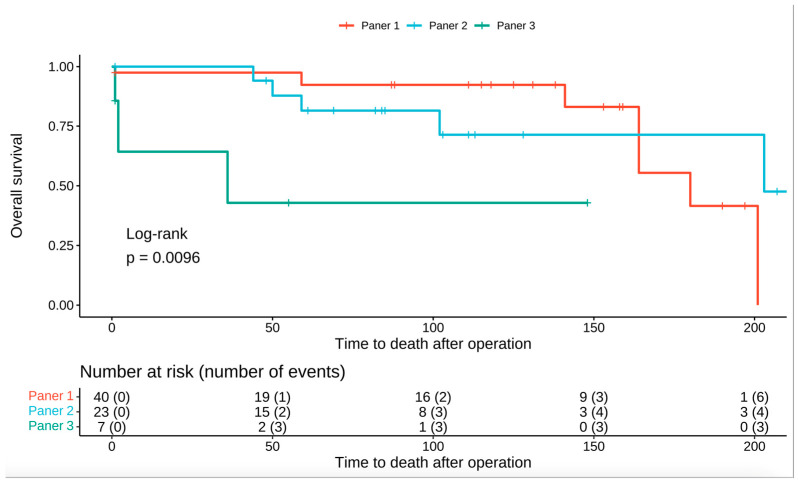
Kaplan–Meier curve showing overall survival for the Paner 3-tier grading system.

Male gender was associated with a 51% higher risk of dying; however, this was not statistically significant (HR: 1.51; 95% CI 0.42–5.35; *p* = 0.5). The risk of death was almost five times higher for patients that were classified as Grade 4 in contrast to patients that were categorized as Grade 1 in the Avulova grading system (HR: 5.83; 95% CI, 1.37–24.7; *p* = 0.017). Moreover the same applies for patients that were graded as Grade 3 in the Paner grading system, having almost a 5 times higher risk of dying than patients that were graded as grade 1 (HR: 5.82; 95% CI, 1.37–24.7; *p* = 0.017). Overall survival for the Avulova and Paner grading systems is illustrated in Figure 2 and Figure 3, respectively. As far as the IHC is concerned, mTOR expression was associated with an HR of 8.57 (95% CI, 1.91–38.5; *p* = 0.005) and CYLD expression was associated with an HR of 17.3 (95% CI, 1.57–192; *p* = 0.02). The hazard ratios for clinical and pathological parameters are illustrated in Table 4.

## 4. Discussion

As RCC represents 2–3% of all cancers, ChRCC is a rare and distinct biological subtype that shares little with other RCC subtypes [3,23]. Usually, the clinical course of ChRCC is less aggressive given that it is usually localized; thus, radical or partial nephrectomy offer excellent oncologic results. Typically, patients with ChRCC tend to be younger, with a female predominance and the majority present with a lower T stage (T1 or T2) and rarely with sarcomatoid differentiation [24]. As such, they have a better prognosis compared to clear cell RCC, with data from the Italian SATURN project (including 291 patients) suggesting an even better 5- and 10-year CSS, reaching 93% and 88.9%, respectively [23]. However, occasionally, patients will present with advanced disease, while the incidence of metastatic cases can reach 6–7% [23]. Tumor grade is one of the most important prognostic parameters of renal cancer. Given that a small but significant group of patients will present with aggressive disease, grading of ChRCC is necessary.

Given that ChRCC tumors are characterized by innate atypia, they cannot be graded according to traditional grading systems, such as the WHO/ISUP or the Fuhrman grading systems [5,6,8]. As a result, various efforts have been directed at grading ChRCC tumors, aiming to discount the atypical cell characteristics of ChRCC tumors. The latest four-tier classification system that was proposed by Avulova et al. has shown promising results [13]. In their study, the authors incorporated coagulative tumor necrosis and, as a result, they expanded the previous three-tier system that was proposed by Paner et al. into a four-tier system, thus improving its prognostic ability [8,13]. Coagulative tumor necrosis has been correlated with poor prognosis in many different studies [9,10,11,12]. In our study, we found that the risk of death was higher in patients that were categorized as Grade 3 in the Paner system or Grade 4 in the Avulova system. However, the presence of necrosis in our histology specimens was not correlated with a poorer clinical outcome, as the univariate analysis has shown. Another independent prognostic factor that is associated with poor survival is the presence of sarcomatoid differentiation, and these cases should be closely monitored [9,10,11,12,23,25]. In our study, there were three patients with sarcomatoid differentiation; two of them died because of the disease, confirming its poor prognostic outcome. Tumor size and pathologic T stage have been correlated with poor survival, although this was not shown in our study [6,9,11,12,26]. Possibly because there were not many cancer-specific events in our study (such as metastasis- or cancer-related death), prognostic parameters that are often linked to worse outcomes, they were not demonstrated to have that association in our study. Finally, we demonstrated that age is associated with worse overall survival, as other studies have shown; however, this is not due to a worse prognosis other than the possibility of dying from unrelated causes [6,9].

Patients with BHD syndrome, are at risk of developing multifocal, bilateral renal tumors with hybrid oncocytic tumors and ChRCC being the most frequent types [27]. It has been postulated that because of inactivating mutations of the folliculin gene, this leads to the development of tumors with an upregulated P13/Akt/mTOR axis [15]. Further molecular characterization of ChRCC tumors revealed various alterations in the mTOR pathway that were correlated with poorer survival [16]. In our study, positive mTOR staining was negatively correlated with survival, with the rate of positive staining increasing by more than two-fold, as it was positive in 86% (12 out of 15) of patients that eventually died vs. 41% (22 out of 55) of patients that survived. Although there was no molecular characterization of these tumors, patients with mTOR pathway alterations have shown a good response to mTOR-targeted therapies, and this association should be further explored in the future, given the available evidence in various renal tumors [28,29]. As far as CYLD IHC expression is concerned, low immunoreactive scores were found in the ChRCC tumors that were studied showing a possible downregulation of the CYLD tumor-suppressor protein. It has been demonstrated previously that CYLD mRNA has been downregulated in cancerous ccRCC tissue, in which it is normally expressed in normal kidney tissue, showing its possible correlation in the tumorigenesis [21]. Given the role of the CYLD tumor-suppressor protein in various signaling pathways, such as the nuclear factor kappa B (NFkΒ) and the mitogen-activated protein kinase (MAPK) pathways, linking it with various cancers, it could be involved in the oncogenesis process of ChRCC tumors [20,30]. However, this association needs to be further explored, given its therapeutic potential, especially given the current envy of available treatment options [17].

In our study we aimed to evaluate different clinical and histopathologic features in patients diagnosed with ChRCC from two different tertiary hospitals; however, there are some limitations that need to be noted. First, this was a retrospective study, incorporating databases from two distinct pathology departments, which has the potential to introduce many confounders. Moreover, even though the study was conducted at a tertiary center, the paucity of cancer-specific events decreased the power of the survival analysis, as it is specifically related to the number of events rather than participants. As a result, cancer-specific survival analyses could not be performed. Cancer-specific survival or progression are much more important clinical markers, as they would help us to identify this small subgroup that has an increased risk of developing metastasis. Consequently, we could not perform multivariate analysis due to the small number of deaths. In this regard, prospective multicenter studies are needed that will have a long follow-up period in order to clarify the findings around ChRCC.

## 5. Conclusions

According to our study, in which 4% of the patients with ChRCC died from the disease, these patients have a low risk of both cancer-related death and disease progression, which is in accordance with the current literature. The Avulova grading system has been found to be positively correlated with OS, as shown in previous studies further cementing its utility as a proper grading system for the ChRCC subtype. Furthermore, the presence of an elevated mTOR was associated with worse OS, underlining the role of the P13/Akt/mTOR axis in this specific disease. Larger studies, probably as a multi-institutional collaboration, are needed in order to accumulate more “tumor-related” events so that we can validate or further clarify the available data around this rare disease entity.

## Figures and Tables

**Table 1:** General characteristics of the patients.

Characteristic	Number (%) or Mean (±SD)
Total patients	72
Follow-up duration	44–222 months
Followed for >5 years	84%
Followed for >10 years	54%
Male	74%
Age (years)	60.3 (±15.1)
Tumor size (mm)	10–230 (62.6 ± 44.0)
Radical nephrectomy	69%
Metastatic at operation	3 (4%)

**Table 2:** Univariate analysis of the clinical parameters associated with death.

Characteristic	Overall (n = 70)	Living (n = 55)	Death (n = 15)	*p*-Value
Males	52 (74%)	40 (73%)	12 (80%)	0.8
Age (years)	60.3 ± 15.1	58.3 ± 15.7	67.4 ± 10.2	0.014
Type of operation				0.018
- Nephrectomy	48 (69%)	42 (76%)	6 (40%)	
- Partial nephrectomy	22 (31%)	13 (24%)	9 (60%)	
Greatest dimension (mm)	62.6 ± 44.0	66.4 ± 46.7	49.1 ± 30.3	0.10
Follow-up duration (months)	67.4 ± 71.1	60.3 ± 68.9	93.8 ± 75.0	0.13

**Table 3:** Univariate analysis of the pathological parameters associated with death.

Characteristic	Overall (n = 70)	Living (n = 55)	Death (n = 15)	*p*-Value
Greatest dimension (mm)	62.6 ± 44.0	66.4 ± 46.7	49.1 ± 30.3	0.10
T Stage				0.6
- T1a	29 (41%)	22 (40%)	7 (47%)	
- T1b	13 (19%)	9 (16%)	4 (27%)	
- T2a	6 (8.6%)	6 (11%)	0 (0%)	
- T2b	9 (13%)	8 (15%)	1 (6.7%)	
- T3a	12 (17%)	9 (16%)	3 (20%)	
- T3b	1 (1.4%)	1 (1.8%)	0 (0%)	
Surgical margins				0.12
- No data	2 (2.9%)	2 (3.6%)	0 (0%)	
- Negative	65 (93%)	52 (95%)	13 (87%)	
- Positive	3 (4.3%)	1 (1.8%)	2 (13%)	
- 1	40 (57%)	33 (60%)	7 (47%)	
- 2	23 (33%)	18 (33%)	5 (33%)	
- 3	7 (10%)	4 (7.3%)	3 (20%)	
Grade (Avulova)				0.5
- 1	40 (57%)	33 (60%)	7 (47%)	
- 2	15 (21%)	12 (22%)	3 (20%)	
- 3	8 (11%)	6 (11%)	2 (13%)	
- 4	7 (10%)	4 (7.3%)	3 (20%)	
Necrosis	20 (29%)	15 (27%)	5 (33%)	0.9
Sarcomatoid change	3 (4.3%)	1 (1.8%)	2 (13%)	0.2
Lymphovascular invasion	5 (7.1%)	3 (5.5%)	2 (13%)	0.6
CK7	63 (90%)	49 (89%)	14 (93%)	>0.9
Vimentin	8 (11%)	6 (11%)	2 (13%)	>0.9
CD10	22 (31%)	17 (31%)	5 (33%)	>0.9
ki67 expression	46 (66%)	36 (65%)	10 (67%)	>0.9
CD117 (c-kit)	60 (86%)	48 (87%)	12 (80%)	0.8
mTOR Staining	34 (50%)	22 (41%)	12 (86%)	0.007
Cyld IRS				0.004
- 0	59 (87%)	49 (92%)	10 (67%)	
- 3	4 (5.9%)	3 (5.7%)	1 (6.7%)	
- 4	3 (4.4%)	0 (0%)	3 (20%)	
- 8	1 (1.5%)	0 (0%)	1 (6.7%)	
- 12	1 (1.5%)	1 (1.9%)	0 (0%)	
Cyld Staining	2 (2.9%)	1 (1.9%)	1 (6.7%)	>0.9

**Table 4:** Hazard ratios for clinical and pathological parameters.

Characteristic	HR	95% CI	*p*-Value
Male	1.51	0.42, 5.35	0.5
Patient age	1.05	1.00, 1.11	0.049
Neoplasm Subtype			
- Classic	—	—	
- Eosinophillic	0.98	0.30, 3.20	>0.9
- Mixed	0.17	0.02, 1.59	0.12
Grade (ISUP 2013)			
- Grade 1	—	—	
- Grade 2	0.37	0.06, 2.47	0.3
- Grade 3	0.27	0.06, 1.30	0.10
- Grade 4	2.73	0.54, 13.8	0.2
Grade (Paner)			
- Grade 1	—	—	
- Grade 2	0.76	0.21, 2.68	0.7
- Grade 3	5.82	1.37, 24.7	0.017
Grade (Avulova)			
- Grade 1	—	—	
- Grade 2	0.71	0.16, 3.06	0.6
- Grade 3	0.84	0.16, 4.35	0.8
- Grade 4	5.83	1.37, 24.7	0.017
mTOR staining	8.57	1.91, 38.5	0.005
Cyld staining	17.3	1.57, 192	0.020

## Data Availability

The data presented in this study are available on request from the corresponding author.
